# A combined immune–nutritional score as a prognostic indicator in neoadjuvant-treated esophageal squamous cell carcinoma

**DOI:** 10.3389/fimmu.2026.1764277

**Published:** 2026-02-26

**Authors:** Qichang Xie, Junpeng Zhan, Xiaolin Ye, Cheng Huang, Chun Chen, Bin Zheng, Guobing Xu, Zhang Yang, Yong Zhu

**Affiliations:** 1 Department of Thoracic Surgery, Fujian Medical University Union Hospital, Fuzhou, China; 2 Key Laboratory of Cardio-Thoracic Surgery (Fujian Medical University), Fuzhou, China; 3 Clinical Research Center for Thoracic Tumors of Fujian Province, Fuzhou, China; 4 Department of Gynecology, Zhangzhou Hospital, Fujian, Zhangzhou, China

**Keywords:** controlling nutritional status score, esophageal squamous cell carcinoma, Gustave Roussy immune score, neoadjuvant therapy, nomogram, prognosis

## Abstract

**Background:**

Reliable biomarkers that predict treatment response and long-term outcomes in neoadjuvant-treated esophageal squamous cell carcinoma (ESCC) remain limited. The Gustave Roussy Immune (GRIm) score and the Controlling Nutritional Status (CONUT) score respectively reflect systemic inflammation and nutritional status. This study evaluated whether integrating these indices (GRIm–CONUT) improves prognostic prediction in ESCC.

**Methods:**

We retrospectively analyzed 216 patients with resectable ESCC who received neoadjuvant chemotherapy, chemoradiotherapy, or chemoimmunotherapy followed by curative (R0) esophagectomy between June 2016 and December 2021. GRIm and CONUT scores were calculated from pre-treatment laboratory parameters, and patients were classified into three GRIm–CONUT categories (0, 1, 2). Logistic regression identified independent predictors of pathological complete response (pCR). Overall survival (OS) and recurrence-free survival (RFS) were assessed using Kaplan–Meier and Cox proportional hazards models. A nomogram incorporating GRIm–CONUT and ypTNM staging was developed and validated using bootstrap resampling.

**Results:**

Higher GRIm–CONUT scores were significantly associated with lower pCR rates (p < 0.01) and poorer OS and RFS (all p < 0.001). Multivariate logistic regression confirmed GRIm–CONUT score, cN stage, and neoadjuvant regimen as independent predictors of pCR. In multivariate Cox analysis, GRIm–CONUT and ypN stage remained independent predictors of OS, while GRIm–CONUT, ypT stage, and ypN stage independently predicted RFS. A GRIm–CONUT–based nomogram demonstrated superior discrimination (C-index 0.717 vs. 0.659 for ypTNM) and offered greater clinical net benefit in decision-curve analysis.

**Conclusions:**

The GRIm–CONUT composite score is an independent predictor of pCR, OS, and RFS in ESCC patients undergoing neoadjuvant therapy and surgery. As an inexpensive and readily obtainable biomarker, it enables more accurate prognostic stratification and may support personalized perioperative management. Prospective multicenter validation is warranted.

## 1 Introduction

Esophageal squamous cell carcinoma (ESCC) remains a major global health burden, with persistently low long-term survival despite advances in multimodal therapy (1, 2). For patients with locally advanced disease, neoadjuvant chemotherapy, chemoradiotherapy, or chemoimmunotherapy followed by curative esophagectomy has become the standard of care, improve tumor downstaging and resection rates (3). However, responses to neoadjuvant treatment vary widely. Some patients achieve pathologic complete response (pCR) and derive substantial survival benefit, whereas others exhibit limited tumor regression and early recurrence (4, 5). Because assessment of treatment efficacy is traditionally based on postoperative pathology, clinicians currently lack reliable pretreatment biomarkers to stratify patients by expected treatment response and prognosis. Identifying accessible, reproducible, and cost-effective markers capable of guiding individualized treatment strategies remains an important unmet need.

Systemic inflammation and nutritional status are now recognized as key determinants of cancer progression, treatment tolerance, and postoperative outcomes (6). Inflammatory cytokines promote tumor proliferation, immune evasion, and metastatic potential, while malnutrition and lymphocyte depletion impair antitumor immunity and reduce the host’s physiologic reserve (7, 8). Biomarkers reflecting these host-related factors have shown prognostic relevance across several malignancies. The Gustave Roussy Immune (GRIm) score—composed of neutrophil-to-lymphocyte ratio (NLR), serum albumin, and lactate dehydrogenase (LDH)—captures systemic inflammatory and immune status and has shown prognostic utility patients receiving antitumor therapy (9–11). The Controlling Nutritional Status (CONUT) score—based on serum albumin, lymphocyte count, and total cholesterol, is an objective index of nutritional and immune-nutritional status and has similarly been associated with outcomes in various cancers, including ESCC (12, 13).

Although both GRIm and CONUT independently reflect important aspects of host biology, they assess complementary domains: GRIm emphasizes systemic inflammatory and immune activation, whereas CONUT reflects nutritional reserve and lipid metabolism. Integrating these indices may therefore offer a more comprehensive assessment of the immune–nutritional condition than either marker alone. However, the prognostic significance of a combined GRIm–CONUT score in ESCC patients undergoing neoadjuvant therapy has not been systematically evaluated.

In this study, we investigated whether the pretreatment GRIm–CONUT composite score predicts pathologic response and long-term survival in ESCC patients treated with neoadjuvant therapy followed by surgery. We further compared its prognostic performance with individual GRIm and CONUT scores and constructed a postoperative nomogram integrating GRIm–CONUT with ypTNM staging. We hypothesized that the combined immune–nutritional index would provide superior prognostic discrimination and offer practical value for individualized treatment planning and postoperative management.

## 2 Methods

### 2.1 Patients

This retrospective study enrolled patients who received neoadjuvant therapy followed by radical esophagectomy at our institution between June 2016 and December 2021. The inclusion criteria were as follows: (1) age between 18 and 75 years; (2) pathological confirmation of esophageal squamous cell carcinoma (ESCC); and (3) clinical stage II–IVA according to the American Joint Committee on Cancer (AJCC) 8th edition TNM staging system. Patients were excluded based on the following criteria: (1) concurrent other malignancies or ongoing anticancer therapy for other cancers; (2) postoperative pathology indicating mixed histological types; (3) non-R0 resection; (4) incomplete pre-treatment laboratory data; (5) presence of distant metastasis; or (6) lack of postoperative follow-up information. A total of 216 patients were included in the final analysis. The study protocol was approved by the Ethics Committee of Fujian Medical University Union Hospital (Approval No. 2025KY081) and was conducted in accordance with the principles of the Declaration of Helsinki and its subsequent amendments. Given the retrospective nature of the study, a waiver of informed consent was granted.

### 2.2 Procedures

We retrospectively collected clinical data from patient cases within one week prior to neoadjuvant therapy initiation. The data encompassed baseline characteristics—including sex, age, body mass index (BMI), comorbidities, smoking history, and alcohol consumption—as well as hematological parameters such as hemoglobin, lactate dehydrogenase, neutrophil-to-lymphocyte ratio(NLR), serum albumin, total lymphocyte count, and total cholesterol. Tumor-related information was also recorded, covering tumor location, neoadjuvant treatment regimen, clinical T and N stages, postoperative pathological T and N stages, and tumor regression score.

### 2.3 Definition of GRIm-CONUT score

**Figure 1:**
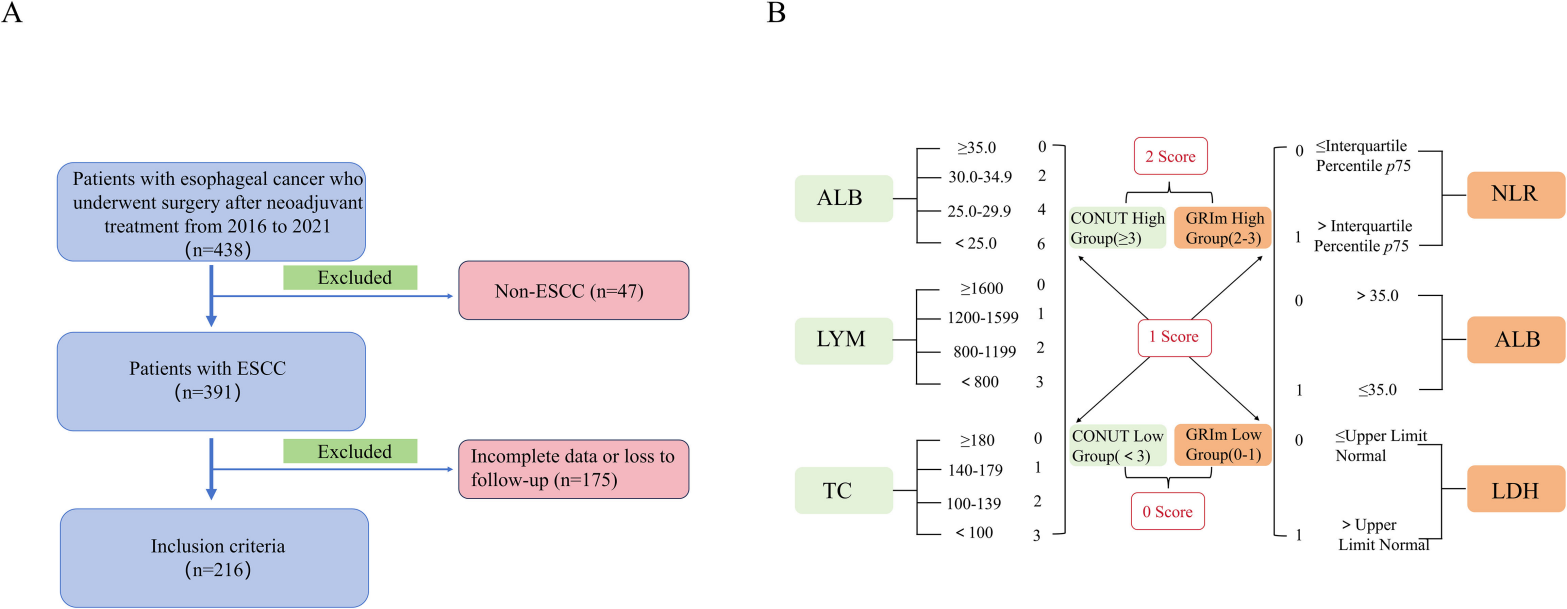
**(A)** Patient eligibility screening procedure; **(B)** Definition and composition of the GRIm-CONUT Score. ESCC, esophageal squamous cell carcinoma; ALB, albumin; LYM, lymphocyte; TC, total cholesterol; NLR, neutrophil-to-lymphocyte ratio; LDH, lactate dehydrogenase; CONUT, controlling nutritional status score; GRIm, Gustave Roussy immune score.

GRIm was calculated using the following three variables: LDH (within the normal range: 0 vs. > Upper limit of normal (ULN) for each center, +1 for 245 U/L in our hospital), ALB (≥ 35 g/L: 0 vs. < 35 g/L: +1), and NLR (≤ p 75: 0 vs. > p 75: +1). Patients were divided into two groups: a high group (2 or 3 points) and a low group (0 or 1 point) (reference). The CONUT score consisted of LYM, TC, and ALB. Patients were divided into two groups based on their CONUT score: a low group (< 3 points) and a high group (≥ 3 points). Based on this, the study subjects were further divided into three groups: a GRIm-CONUT score of 2 (high GRIm and high CONUT), a GRIm-CONUT score of 1 (high GRIm and low CONUT or low GRIm and high CONUT), and a GRIm-CONUT score of 0 (low GRIm and low CONUT). Specific definitions are shown in **Figure 1**. A score of 0 indicates essentially normal nutritional and inflammatory status. A score of 1 reflects a mild, single-system abnormality, which is clinically common and generally manageable. A score of 2 signifies a more severe derangement.

### 2.4 Treatment protocol

Neoadjuvant regimens included: neoadjuvant chemotherapy (2–4 cycles of paclitaxel or albumin-bound paclitaxel combined with platinum-based regimens, every 3 weeks), neoadjuvant chemoradiotherapy (same chemotherapy regimen, with a radiotherapy dose of 40–50 Gy/23F), and neoadjuvant chemoimmunotherapy (same chemotherapy regimen, combined with immunotherapy including pembrolizumab, camrelizumab, or sintilimab). All patients underwent radical surgery within 4–6 weeks after the completion of neoadjuvant therapy, either thoracoscopic or laparoscopic combined with da Vinci robotic-assisted McKeown or Ivor Lewis esophagectomy.

### 2.5 Follow-up

All patients included in the study underwent systematic and regular follow-up. Follow-up primarily included survival status, disease recurrence and metastasis, and treatment status. Patients were followed up every 3 months for the first 2 years post-surgery, every 6 months from years 3 to 5, and annually thereafter. Monitoring methods included hematological markers, CT scans of the neck, chest, and abdomen, PET/CT, and endoscopy. The primary endpoint was overall survival(OS), defined as the period from the date of surgery to the last follow-up or the occurrence of the endpoint event (death). The secondary endpoint was recurrence-free survival (RFS), defined as the time from surgery to the first documented recurrence, distant metastasis, death from any cause, or the last confirmed recurrence-free follow-up.

### 2.6 Data analysis

This study used R 4.5.0 and SPSS 20 for data analysis. Continuous variables between groups were analyzed using t-tests or Mann-Whitney U tests, and dichotomous variables between groups were identified using chi-square tests or Fisher’s exact tests. OS and RFS were assessed using Kaplan-Müller’s method (KM) analysis, and curve differences were analyzed using log-rank tests. Univariate and multivariate Cox regression analysis was used to identify risk factors predicting OS and RFS; variables with p < 0.05 were included in multivariate regression analysis. Models were constructed based on the multivariate regression coefficients to predict OS. A nomogram model was constructed using the rms package in R for visualization, and the area under the ROC curve (AUC), time-dependent ROC curves, calibration curves, and decision curve analysis (DCA) were constructed using the pROC, riskRegression, rms, and dcurves packages to evaluate the performance of the predictive model. No external validation was designed; internal validation was performed using Bootstrap with 1000 iterations.

## 3 Result

### 3.1 Patient characteristics

A total of 216 patients with locally advanced ESCC who received neoadjuvant therapy followed by curative surgery were included in the analysis. Based on pretreatment laboratory indices, patients were categorized into three GRIm–CONUT groups: score 0 (n = 118, 54.63%), score 1 (n = 79, 36.57%), and score 2 (n = 19, 8.80%).Baseline demographic and clinical characteristics are summarized in **Table 1**. Significant differences among the three GRIm–CONUT groups were observed in sex distribution (*P* = 0.015). In contrast, age, BMI, smoking status, alcohol consumption, and comorbidities—including hypertension, diabetes, and heart disease—did not differ significantly across groups (all *P* > 0.05).

**Table 1:** Baseline characteristics table of the study population.

Variables	Total (n = 216)	GRIm-CONUT=0 (n = 118)	GRIm-CONUT=1 (n = 79)	GRIm-CONUT=2 (n = 19)	*P*
Gender, n(%)					0.015
Male	162 (75.000)	86 (72.881)	66 (83.544)	10 (52.632)	
Female	54 (25.000)	32 (27.119)	13 (16.456)	9 (47.368)	
AGE, n(%)					0.461
≤60	103 (47.685)	53 (44.915)	42 (53.165)	8 (42.105)	
>60	113 (52.315)	65 (55.085)	37 (46.835)	11 (57.895)	
Smoking, n(%)					0.064
No	101 (46.759)	57 (48.305)	31 (39.241)	13 (68.421)	
Yes	115 (53.241)	61 (51.695)	48 (60.759)	6 (31.579)	
Drinking, n(%)					0.279
No	111 (51.389)	65 (55.085)	35 (44.304)	11 (57.895)	
Yes	105 (48.611)	53 (44.915)	44 (55.696)	8 (42.105)	
Hypertension, n(%)					0.232
No	192 (88.889)	108 (91.525)	69 (87.342)	15 (78.947)	
Yes	24 (11.111)	10 (8.475)	10 (12.658)	4 (21.053)	
Diabetes, n(%)					0.171
No	196 (90.741)	109 (92.373)	72 (91.139)	15 (78.947)	
Yes	20 (9.259)	9 (7.627)	7 (8.861)	4 (21.053)	
HD, n(%)					0.820
No	210 (97.222)	115 (97.458)	76 (96.203)	19 (100.000)	
Yes	6 (2.778)	3 (2.542)	3 (3.797)	0 (0.00)	
ASA, n(%)					0.529
I	14 (6.481)	7 (5.932)	5 (6.329)	2 (10.526)	
II	176 (81.481)	93 (78.814)	67 (84.810)	16 (84.211)	
III	26 (12.037)	18 (15.254)	7 (8.861)	1 (5.263)	
BMI, n(%)					0.279
<18	20 (9.259)	11 (9.322)	5 (6.329)	4 (21.053)	
18-24	154 (71.296)	82 (69.492)	61 (77.215)	11 (57.895)	
>24	42 (19.444)	25 (21.186)	13 (16.456)	4 (21.053)	
cT, n(%)					0.068
1	16 (7.407)	8 (6.780)	6 (7.595)	2 (10.526)	
2	71 (32.870)	35 (29.661)	27 (34.177)	9 (47.368)	
3	81 (37.500)	55 (46.610)	22 (27.848)	4 (21.053)	
4	48 (22.222)	20 (16.949)	24 (30.380)	4 (21.053)	
cN, n(%)					0.019
0	43 (19.907)	30 (25.424)	13 (16.456)	0 (0.00)	
1	45 (20.833)	23 (19.492)	19 (24.051)	3 (15.789)	
2	83 (38.426)	47 (39.831)	29 (36.709)	7 (36.842)	
3	45 (20.833)	18 (15.254)	18 (22.785)	9 (47.368)	
ypT, n(%)					0.023*
0	24 (11.111)	20 (16.949)	2 (2.532)	2 (10.526)	
1	30 (13.889)	17 (14.407)	12 (15.190)	1 (5.263)	
2	39 (18.056)	20 (16.949)	17 (21.519)	2 (10.526)	
3	110 (50.926)	57 (48.305)	41 (51.899)	12 (63.158)	
4	13 (6.019)	4 (3.390)	7 (8.861)	2 (10.526)	
ypN, n(%)					0.022
0	102 (47.222)	65 (55.085)	32 (40.506)	5 (26.316)	
1	57 (26.389)	32 (27.119)	18 (22.785)	7 (36.842)	
2	47 (21.759)	16 (13.559)	25 (31.646)	6 (31.579)	
3	10 (4.630)	5 (4.237)	4 (5.063)	1 (5.263)	
TRG, n(%)					0.052
0	24 (11.111)	20 (16.949)	2 (2.532)	2 (10.526)	
1	63 (29.167)	32 (27.119)	25 (31.646)	6 (31.579)	
2	75 (34.722)	41 (34.746)	30 (37.975)	4 (21.053)	
3	54 (25.000)	25 (21.186)	22 (27.848)	7 (36.842)	
Location, n(%)					0.317
Upper	23 (10.648)	12 (10.169)	11 (13.924)	0 (0.00)	
Middle	131 (60.648)	71 (60.169)	49 (62.025)	11 (57.895)	
Lower	62 (28.704)	35 (29.661)	19 (24.051)	8 (42.105)	
Method, n(%)					0.807
nICT	30 (13.889)	16 (13.559)	11 (13.924)	3 (15.789)	
nCRT	43 (19.907)	25 (21.186)	13 (16.456)	5 (26.316)	
nCT	143 (66.204)	77 (65.254)	55 (69.620)	11 (57.895)	

HD, Heart disease; ASA, American Society of Anesthesiologists physical status classification; BMI, Body Mass Index; cT, cT stage; cN, cN stage; ypT, ypT stage; ypN, ypN stage; TRG, Tumor Regression Grade; nICT, neoadjuvant immunochemotherapy; nCRT, neoadjuvant chemoradiotherapy; nCT, neoadjuvant chemotherapy.

Regarding tumor-related variables, there was a notable difference in clinical N stage (cN) across the GRIm–CONUT categories (*P* = 0.019). The distribution of clinical T stage (cT) and ypT stage demonstrated observable variation across groups, although ypT showed statistical significance (*P* = 0.023*). Similarly, ypN stage significantly differed among the groups (*P* = 0.022). Tumor location and the choice of neoadjuvant regimen (chemotherapy, chemoradiotherapy, or chemoimmunotherapy) showed no significant differences (*P* > 0.05).

Overall, patients with higher GRIm–CONUT scores tended to present with more advanced nodal disease, while other baseline clinical features were generally comparable among the groups.

### 3.2 Correlation analysis

**Figure 2:**
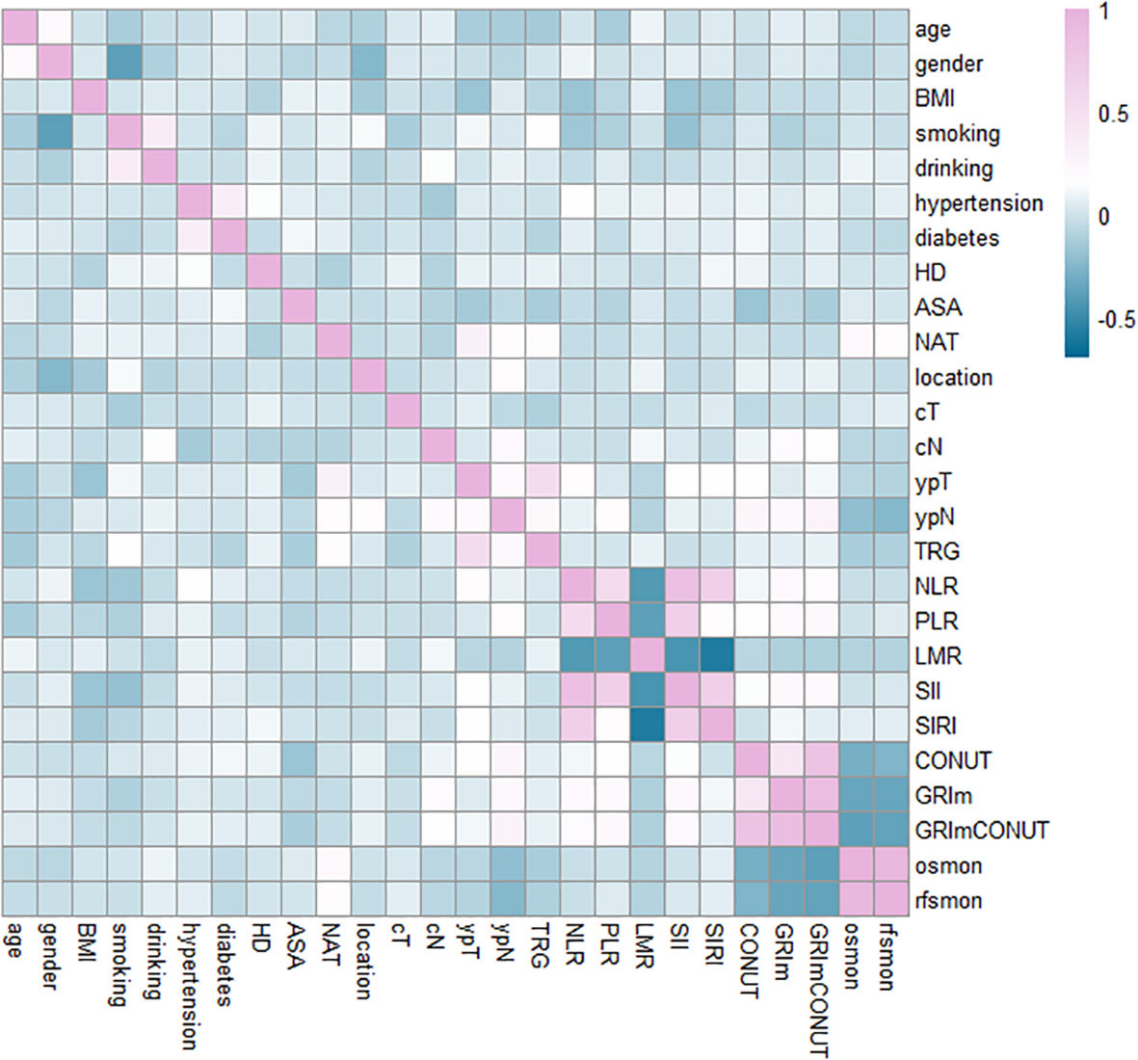
The heatmap shows the correlation between each indicator. The darker the color, the stronger the correlation.

Correlation analysis was performed to evaluate the associations among inflammatory markers, nutritional indicators, clinicopathologic variables, and survival outcomes. As shown in **Figure 2**, both GRIm and CONUT scores demonstrated stronger correlations with overall survival (OS) and recurrence-free survival (RFS) compared with other inflammation- or nutrition-based indices. Notably, although GRIm and CONUT share serum albumin as a common component, their correlation with each other was only moderate, indicating that they capture distinct and complementary aspects of systemic immune–nutritional status.

Importantly, the combined GRIm–CONUT score exhibited a stronger relationship with survival outcomes than either score alone, supporting its value as an integrated prognostic marker. Among clinicopathologic variables, ypN stage showed a significant correlation with both OS and RFS, whereas other tumor characteristics displayed weaker or non-significant associations.

### 3.3 Predictive performance of GRIm-CONUT score

**Figure 3:**
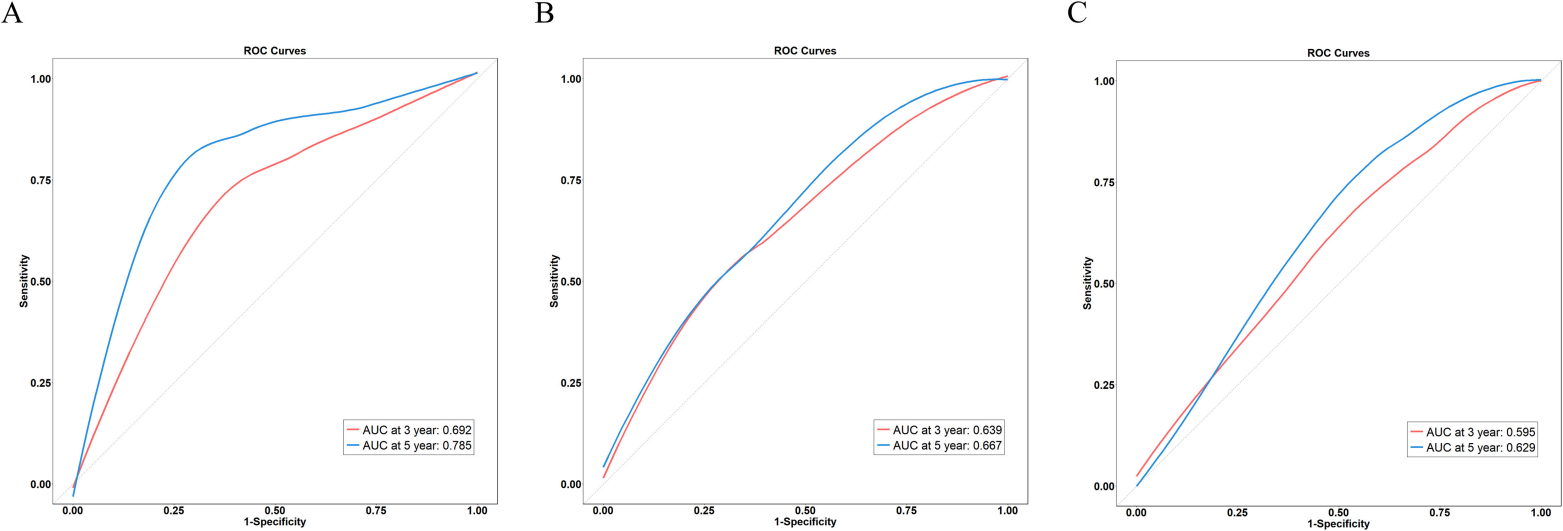
ROC curves of GRIm-CONUT and its independent components. **(A)** GRIm-CONUT, **(B)** GRIm; C, CONUT.

The predictive performance of the GRIm–CONUT score and its individual components was evaluated using ROC curve analysis. As shown in **Figure 3**, the GRIm–CONUT composite demonstrated higher AUC values for predicting OS compared with either the GRIm or CONUT score alone.(GRIm–CONUT vs GRIm or CONUT at 3years:0.692 vs 0.639 or 0.595, P<0.05;GRIm–CONUT vs GRIm or CONUT at 5years:0.785 vs 0.667 or 0.629, P<0.05). These findings indicate that integrating immune-inflammatory and nutritional markers provides a more comprehensive assessment of patient prognosis than using either score in isolation. Moreover, the GRIm–CONUT score outperformed traditional inflammatory markers across multiple follow-up periods, further supporting its robustness as a prognostic tool in the neoadjuvant setting.

### 3.4 Survival analysis

**Figure 4:**
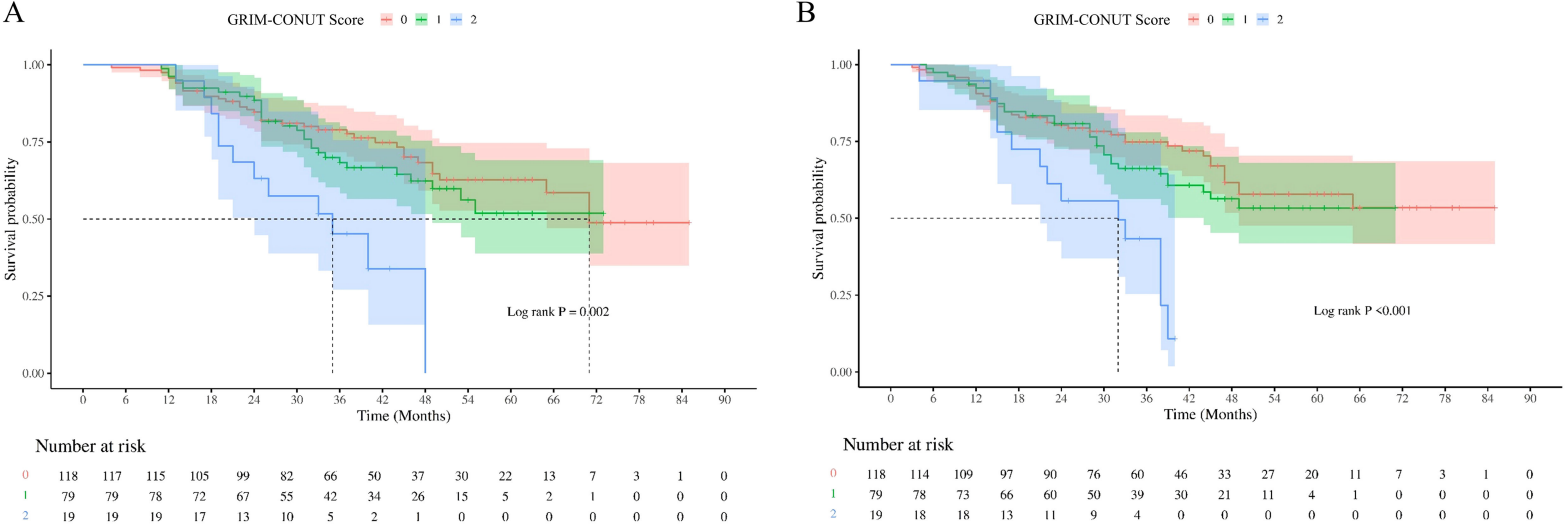
Survival curves based on GRIm-CONUT Score stratification. **(A)** overall survival; **(B)** recurrence free survival.

During a median follow-up of 36.5 months, significant survival differences were observed among the three GRIm–CONUT groups. As illustrated in **Figure 4**, patients with a GRIm–CONUT score of 0 had markedly better OS and RFS than those with scores of 1 or 2. Kaplan–Meier analysis demonstrated a clear stepwise decline in both OS and RFS with increasing GRIm–CONUT categories (*P* = 0.002 for OS; *P* < 0.001 for RFS, log-rank test).

The 5-year OS rates for scores 0, 1, and 2 were 71.19%, 63.29%, and 36.84%, respectively. Corresponding 5-year RFS rates were 67.80%, 60.76%, and 31.58%, indicating substantially higher risks of recurrence and mortality in patients with elevated GRIm–CONUT scores.

These findings support the prognostic relevance of the GRIm–CONUT composite, demonstrating that impaired immune–nutritional status before neoadjuvant therapy is strongly associated with inferior long-term outcomes following curative esophagectomy.

### 3.5 Logistic regression analysis predicts complete pathological remission

Univariate and multivariate logistic regression analyses were performed to identify independent predictors of pathologic complete response (pCR). In the univariate analysis, GRIm–CONUT score, cN stage, and neoadjuvant therapy regimen were significantly associated with pCR.

After adjustment for potential confounders, all three variables—GRIm–CONUT score, cN stage, and neoadjuvant regimen—remained independent predictors in the multivariate model (*P* < 0.05 for all). Patients with a GRIm–CONUT score of 0, those presenting with cN0 disease, and those receiving neoadjuvant chemoimmunotherapy demonstrated notably higher pCR rates (**Table 2**).

**Table 2:** Logistic regression analysis for pathological complete response (pCR).

Variables	Univariate		Multivariate	
	*P*	OR (95%CI)	*P*	OR (95%CI)
GRIm-CONUT Score				
0		1.00 (Reference)		1.00 (Reference)
1	0.013	0.21 (0.06 ~ 0.72)	0.029	0.24 (0.06 ~ 0.87)
2	0.535	0.61 (0.13 ~ 2.87)	0.883	0.88 (0.16~ 4.93)
AGE				
≤60		1.00 (Reference)		
>60	0.141	1.96 (0.80 ~ 4.79)		
Gender				
Male		1.00 (Reference)		
Female	0.618	1.27 (0.50 ~ 3.25)		
Smoking				
No		1.00 (Reference)		
Yes	0.923	1.04 (0.45 ~ 2.44)		
Drinking				
No		1.00 (Reference)		
Yes	0.885	1.06 (0.46 ~ 2.49)		
BMI				
<18		1.00 (Reference)		
18-24	0.227	3.56 (0.45 ~ 27.86)		
>24	0.175	4.5 (0.51 ~ 39.57)		
cT				
1		1.00 (Reference)		
2	0.594	1.82 (0.20 ~ 16.36)		
3	0.142	4.80 (0.59 ~ 38.95)		
4	0.267	3.43 (0.39 ~ 30.18)		
cN				
0		1.00 (Reference)		1.00 (Reference)
1	0.020	0.15 (0.03 ~ 0.75)	0.010	0.10 (0.02 ~ 0.58)
2	0.071	0.40 (0.15 ~ 1.08)	0.030	0.29 (0.09 ~ 0.89)
3	0.038	0.24 (0.06 ~ 0.93)	0.033	0.19 (0.04 ~ 0.88)
Method				
nICT		1.00 (Reference)		1.00 (Reference)
nCRT	0.453	0.64 (0.20 ~ 2.06)	0.105	0.32 (0.08 ~ 1.26)
nCT	0.010	0.25 (0.09 ~ 0.71)	0.002	0.14 (0.04 ~ 0.48)
Location				
Upper		1.00 (Reference)		
Middle	0.827	0.86 (0.23 ~ 3.25)		
Lower	0.655	0.71 (0.16 ~ 3.13)		

BMI, Body Mass Index; cT, cT stage; cN, cN stage; nICT, neoadjuvant immunochemotherapy; nCRT, neoadjuvant chemoradiotherapy; nCT, neoadjuvant chemotherapy.

These findings indicate that pretreatment immune–nutritional status, nodal burden, and treatment modality independently influence the likelihood of achieving pCR following neoadjuvant therapy.

### 3.6 Univariate and multivariate analyses of factors associated with OS

Univariate Cox regression analysis identified several variables significantly associated with OS, including GRIm–CONUT score, ypT stage, ypN stage, and pCR (all *P* < 0.05). Variables with *P* < 0.10 were subsequently included in the multivariate model.

In the multivariate analysis, both GRIm–CONUT score and ypN stage remained independent prognostic factors for OS. Patients with higher GRIm–CONUT scores had a significantly increased risk of mortality (*P* = 0.025), indicating that poorer immune–nutritional status before neoadjuvant therapy is strongly associated with inferior long-term survival. Similarly, advanced ypN stage independently predicted worse OS (*P* < 0.001).

**Figure 5:**
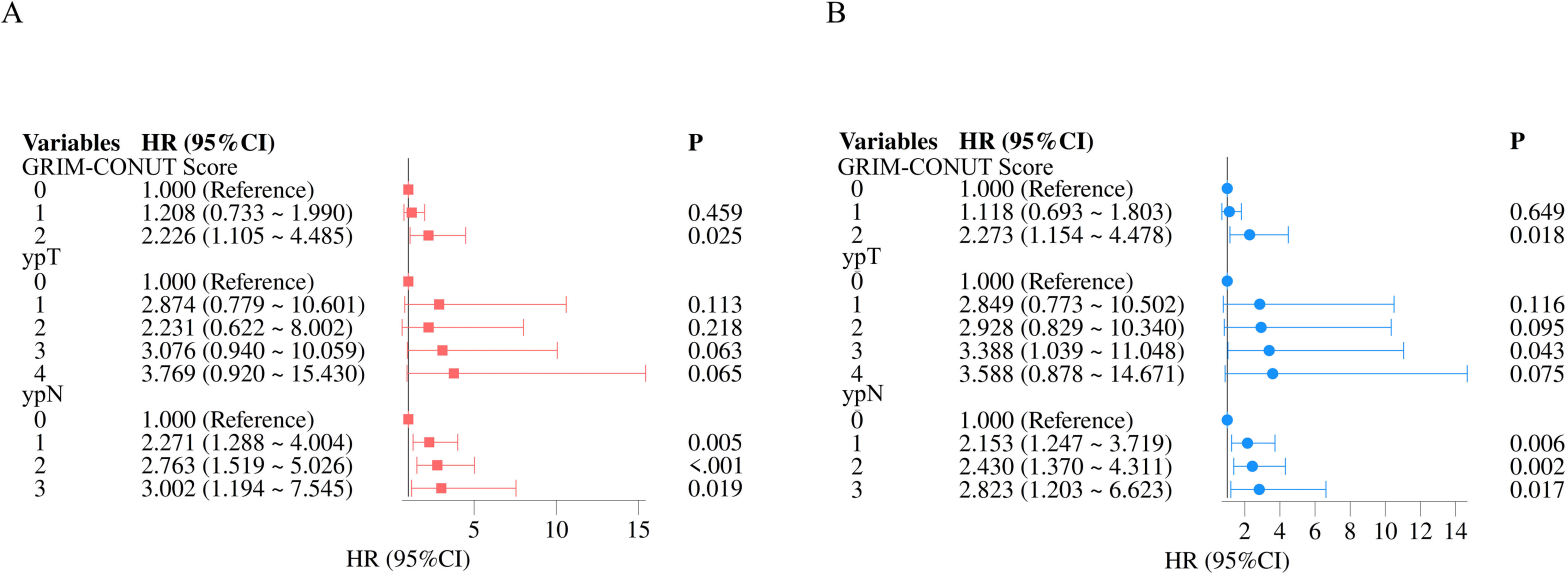
**(A)** Multivariable forest plot for overall survival; **(B)** Multivariable forest plot for recurrence-free survival.

These findings indicate that the GRIm–CONUT composite serves as a robust prognostic indicator for postoperative survival, providing additive prognostic information beyond traditional pathological staging (**Table 3**, **Figure 5A**).

**Table 3:** Univariate and multivariate analyses of factors associated with OS.

Variables	Univariate		Multivariate	
	*P*	HR (95%CI)	*P*	HR (95%CI)
Gender				
Male		1.000 (Reference)		
Female	0.965	0.988 (0.582 ~ 1.678)		
Age				
≤60		1.000 (Reference)		
>60	0.786	0.940 (0.603 ~ 1.467)		
Smoking				
No		1.000 (Reference)		
Yes	0.333	1.250 (0.796 ~ 1.965)		
Drinking				
No		1.000 (Reference)		
Yes	0.414	0.831 (0.532 ~ 1.297)		
Hypertension				
No		1.000 (Reference)		
Yes	0.784	0.903 (0.434 ~ 1.877)		
Diabetes				
No		1.000 (Reference)		
Yes	0.197	1.549 (0.796 ~ 3.013)		
HD				
No		1.000 (Reference)		
Yes	0.612	1.442 (0.351 ~ 5.916)		
ASA				
I		1.000 (Reference)		
II	0.057	0.466 (0.212 ~ 1.023)		
III	0.477	0.712 (0.280 ~ 1.814)		
BMI				
<18		1.000 (Reference)		
18-24	0.942	0.973 (0.464 ~ 2.042)		
>24	0.705	0.846 (0.354 ~ 2.018)		
cT				
1		1.000 (Reference)		
2	0.577	1.310 (0.507 ~ 3.382)		
3	0.910	0.946 (0.363 ~ 2.467)		
4	0.882	1.078 (0.398 ~ 2.925)		
cN				
0		1.000 (Reference)		
1	0.718	1.144 (0.551 ~ 2.373)		
2	0.169	1.544 (0.831 ~ 2.869)		
3	0.990	0.995 (0.465 ~ 2.127)		
ypT				
0		1.000 (Reference)		1.000 (Reference)
1	0.055	3.535 (0.972 ~ 12.851)	0.113	2.874 (0.779 ~ 10.601)
2	0.126	2.688 (0.758 ~ 9.528)	0.218	2.231 (0.622 ~ 8.002)
3	0.019	4.035 (1.255 ~ 12.971)	0.063	3.076 (0.940 ~ 10.059)
4	0.026	4.842 (1.209 ~ 19.398)	0.065	3.769 (0.920 ~ 15.430)
ypN				
0		1.000 (Reference)		1.000 (Reference)
1	0.002	2.619 (1.442 ~ 4.756)	0.005	2.271 (1.288 ~ 4.004)
2	<.001	4.213 (2.330 ~ 7.618)	<.001	2.763 (1.519 ~ 5.026)
3	0.005	3.768 (1.504 ~ 9.441)	0.019	3.002 (1.194 ~ 7.545)
TRG				
0		1.000 (Reference)		
1	0.800	0.892 (0.369 ~ 2.158)		
2	0.476	1.353 (0.589 ~ 3.107)		
3	0.117	1.958 (0.844 ~ 4.541)		
Location				
Upper		1.000 (Reference)		
Middle	0.380	1.466 (0.625 ~ 3.439)		
Lower	0.229	1.728 (0.709 ~ 4.210)		
Method				
nICT		1.000 (Reference)		
nCRT	0.354	1.612 (0.587 ~ 4.429)		
nCT	0.309	1.614 (0.641 ~ 4.063)		
GRIm-CONUT Score				
0		1.000 (Reference)		1.000 (Reference)
1	0.400	1.234 (0.756 ~ 2.015)	0.459	1.208 (0.733 ~ 1.990)
2	<.001	3.171 (1.626 ~ 6.183)	0.025	2.226(1.105 ~ 4.485)

HR, Hazards Ratio; CI, Confidence Interval. HD, Heart disease; ASA, American Society of Anesthesiologists physical status classification; BMI, Body Mass Index; cT, cT stage; cN, cN stage; ypT, ypT stage; ypN, ypN stage; TRG,Tumor Regression Grade; nICT, neoadjuvant immunochemotherapy; nCRT, neoadjuvant chemoradiotherapy; nCT, neoadjuvant chemotherapy.

### 3.7 Univariate and multivariate analyses related to RFS

Univariate Cox regression analysis identified several variables significantly associated with RFS, including GRIm–CONUT score, ypT stage, ypN stage, and pCR (all *P* < 0.05). Variables meeting the threshold of *P* < 0.10 were subsequently incorporated into the multivariate model. In the multivariate analysis, GRIm–CONUT score, ypT stage, and ypN stage remained independent prognostic factors for RFS. Patients with higher GRIm–CONUT scores experienced a significantly higher risk of disease recurrence (*P* = 0.018), while advanced ypT and ypN stages were also independently associated with inferior RFS (*P* < 0.05 for both).

These findings confirm that the GRIm–CONUT composite is an independent predictor of recurrence after neoadjuvant therapy and surgery for ESCC. Incorporating this immune–nutritional index into postoperative prognostic models may improve recurrence risk stratification beyond conventional pathological staging (**Table 4**, **Figure 5B**).

**Table 4:** Univariate and multivariate analyses of factors associated with RFS.

		Univariate		Multivariate
Variables	*P*	HR (95%CI)	*P*	HR (95%CI)
Gender				
Male		1.000 (Reference)		
Female	0.762	0.924 (0.553 ~ 1.544)		
Age				
≤60		1.000 (Reference)		
>60	0.990	0.997 (0.648 ~ 1.535)		
Smoking				
No		1.000 (Reference)		
Yes	0.464	1.176 (0.762 ~ 1.816)		
Drinking				
No		1.000 (Reference)		
Yes	0.528	0.870 (0.565 ~ 1.340)		
Hypertension				
No		1.000 (Reference)		
Yes	0.356	0.695 (0.320 ~ 1.507)		
Diabetes				
No		1.000 (Reference)		
Yes	0.396	1.350 (0.675 ~ 2.702)		
HD				
No		1.000 (Reference)		
Yes	0.775	1.228 (0.300 ~ 5.024)		
ASA				
I		1.000 (Reference)		
II	0.145	0.559 (0.256 ~ 1.223)		
III	0.697	0.831 (0.327 ~ 2.111)		
BMI				
<18		1.000 (Reference)		
18-24	0.741	0.888 (0.439 ~ 1.795)		
>24	0.952	1.025 (0.460 ~ 2.284)		
cT				
1		1.000 (Reference)		
2	0.367	1.542 (0.601 ~ 3.954)		
3	0.789	0.876 (0.334 ~ 2.299)		
4	0.535	1.362 (0.513 ~ 3.614)		
cN				
0		1.000 (Reference)		
1	0.252	1.542 (0.735 ~ 3.233)		
2	0.069	1.835 (0.953 ~ 3.533)		
3	0.235	1.559 (0.749 ~ 3.245)		
ypT				
0		1.000 (Reference)		1.000 (Reference)
1	0.061	3.535 (0.972 ~ 12.851)	0.116	2.849 (0.773 ~ 10.502)
2	0.044	3.568 (1.033 ~ 12.327)	0.095	2.928 (0.829 ~ 10.340)
3	0.014	4.340 (1.352 ~ 13.933)	0.043	3.388 (1.039 ~ 11.048)
4	0.034	4.489 (1.122 ~ 17.967)	0.075	3.588(0.878 ~ 14.671)
ypN				
0		1.000 (Reference)		1.000 (Reference)
1	0.002	2.436 (1.393 ~ 4.258)	0.006	2.153 (1.247 ~ 3.719)
2	<.001	3.365 (1.915 ~ 5.912)	0.002	2.430 (1.370 ~ 4.311)
3	0.003	3.585 (1.537 ~ 8.358)	0.017	2.823 (1.203 ~ 6.623)
TRG				
0		1.000 (Reference)		
1	0.638	1.243 (0.501 ~ 3.083)		
2	0.315	1.569 (0.651 ~ 3.780)		
3	0.081	2.201 (0.907 ~ 5.339)		
Location				
Upper		1.000 (Reference)		
Middle	0.094	2.193 (0.874 ~ 5.498)		
Lower	0.084	2.329 (0.893 ~ 6.075)		
Method				
nICT		1.000 (Reference)		
nCRT	0.125	2.179 (0.805 ~ 5.897)		
nCT	0.171	1.901 (0.758 ~ 4.765)		
GRIm-CONUT Score				
0		1.000 (Reference)		1.000 (Reference)
1	0.412	1.219 (0.759 ~ 1.958)	0.649	1.118 (0.693 ~ 1.803)
2	<.001	3.161 (1.663 ~ 6.009)	0.018	2.273(1.154 ~ 4.478)

HR, Hazards Ratio; CI, Confidence Interval. HD, Heart disease; ASA, American Society of Anesthesiologists physical status classification; BMI, Body Mass Index; cT, cT stage; cN, cN stage; ypT, ypT stage; ypN, ypN stage; TRG,Tumor Regression Grade; nICT, neoadjuvant immunochemotherapy; nCRT, neoadjuvant chemoradiotherapy; nCT, neoadjuvant chemotherapy.

### 3.8 Nomogram construction and performance comparison

**Figure 6:**
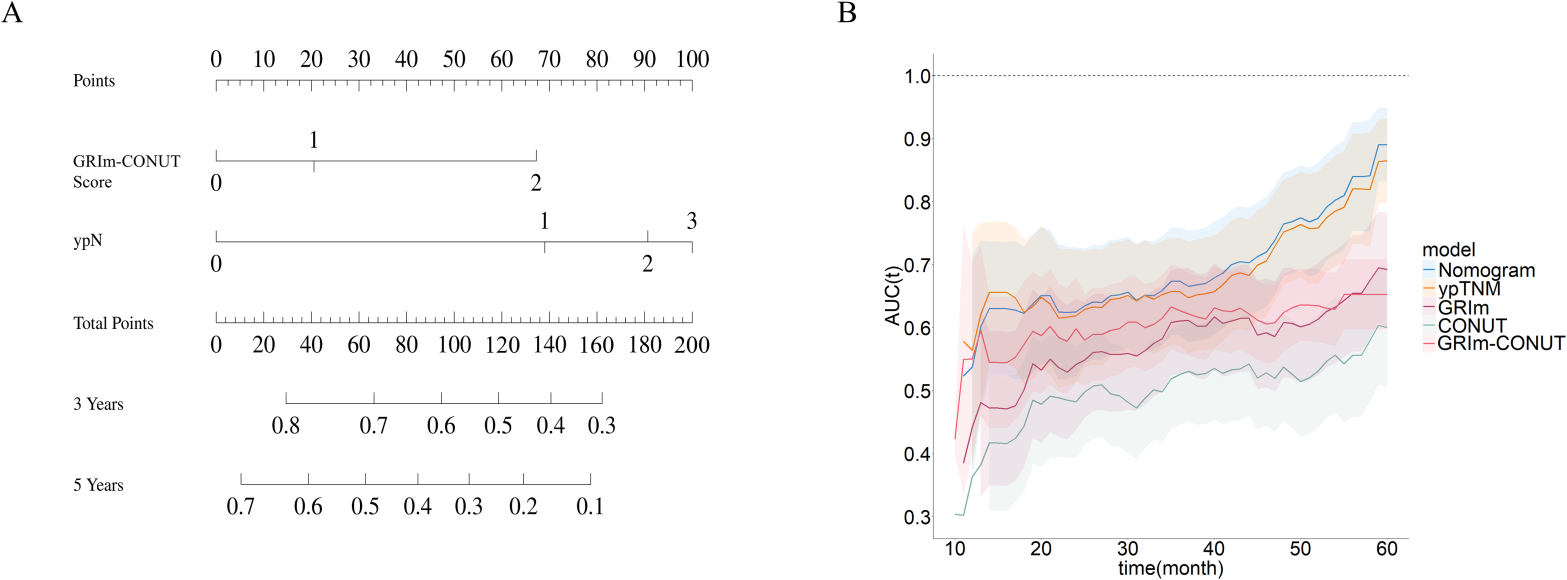
Nomogram for predicting overall survival. **(A)** Construction of a nomogram incorporating GRIm-CONUT score and ypN stage. **(B)** The time-dependent AUC curve shows that during the follow-up period, the predictive performance of the model is superior to that of the traditional ypTNM stage and individual components.

**Figure 7:**
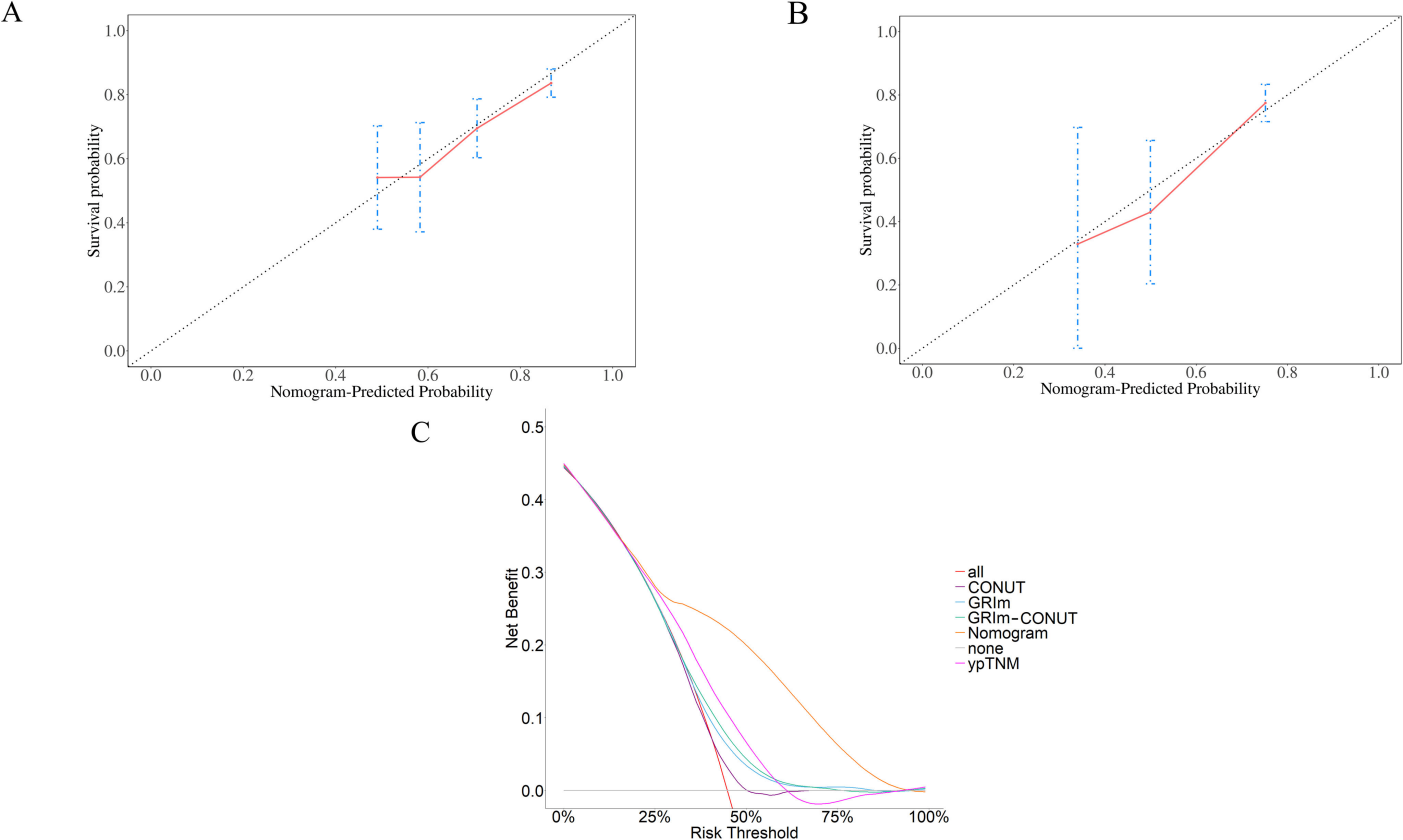
Nomogram performance evaluation. Both the 3-year **(A)** and 5-year **(B)** calibration curves of the nomogram showed good calibration of the model. The decision-curve analysis (DCA) shows that the nomogram yields higher benefits than other models within a reasonable range **(C)**.

Based on the multivariable Cox model, we constructed a prognostic nomogram (nomogram–GRIm–CONUT) that integrates ypTNM stage with the GRIm–CONUT score to estimate postoperative survival in neoadjuvant-treated ESCC(**Figure 6A**). Internal validation using 1,000 bootstrap resamples demonstrated good model stability, with calibration curves showing close agreement between predicted and observed 3- and 5-year survival (**Figures 7A, B**).

The nomogram achieved a higher C-index than the conventional ypTNM staging system (0.717, 95% CI 0.657–0.777 vs 0.659, 95% CI 0.592–0.726), indicating superior discriminative ability(**Figure 6B**). Consistently, time-dependent ROC analyses showed higher AUCs for the nomogram across follow-up, and decision-curve analysis (DCA) demonstrated greater net clinical benefit over a relevant threshold probability range (0.2–1.0) compared with ypTNM alone (**Figure 7C**). Collectively, these results support the added prognostic value of incorporating immune–nutritional status into postoperative risk stratification.

### 3.9 Subgroup regression analysis

**Figure 8:**
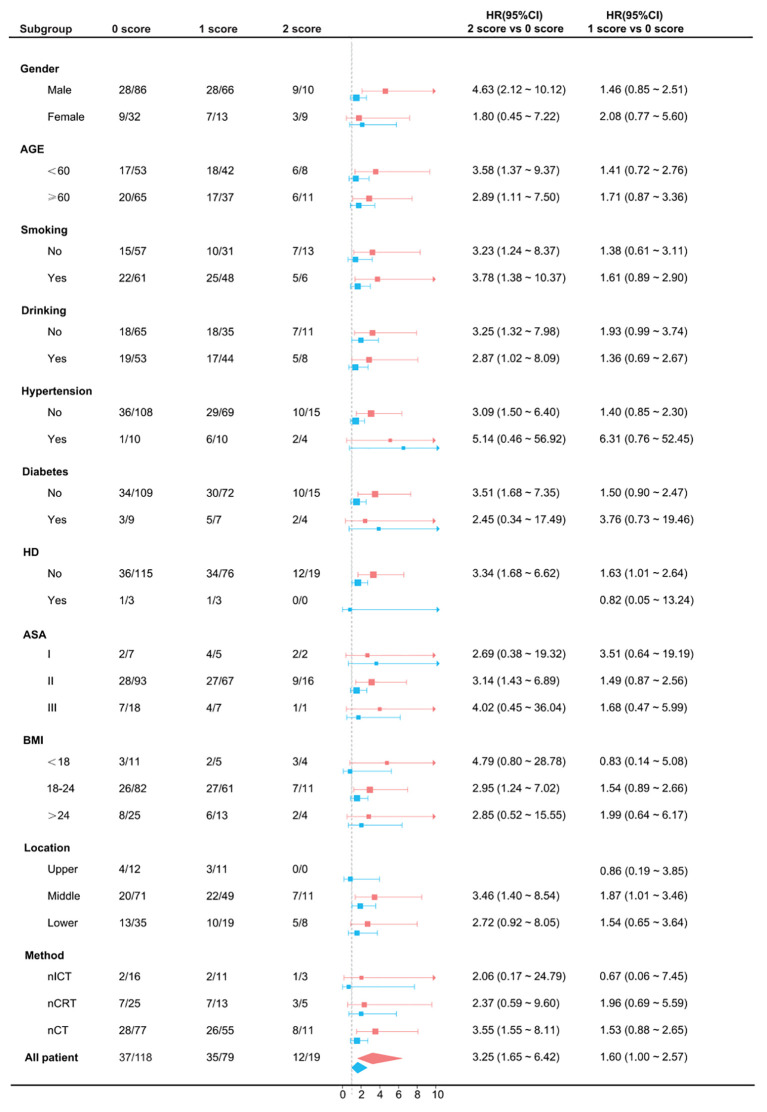
Subgroup analysis across different GRIm-CONUT score categories showed consistency.

Prespecified subgroup analyses demonstrated a consistent association between higher GRIm–CONUT categories and increased risk of death from OS across most strata. The direction and magnitude of effect were broadly concordant, indicating that the prognostic impact of the GRIm–CONUT score was not confined to any single subgroup (**Figure 8**). These findings support the generalizability of the composite immune–nutritional index for postoperative risk stratification in neoadjuvant-treated ESCC.

## 4 Discussion

In this study, we demonstrated that the pretreatment GRIm–CONUT composite score, integrating systemic immune–inflammatory status and nutritional reserve, is a strong predictor of both pathologic response and long-term survival in patients with ESCC undergoing neoadjuvant therapy followed by curative surgery. Higher GRIm–CONUT scores were consistently associated with lower pCR rates, poorer OS and RFS, and a higher risk of disease recurrence. These findings suggest that immune–nutritional impairment prior to treatment may critically influence biological behavior, treatment sensitivity, and postoperative outcomes.

Our results align with growing evidence that the host’s inflammatory and nutritional status plays a central role in cancer progression, treatment efficacy, and immune competence. Previous studies have evaluated the prognostic significance of the GRIm and CONUT scores individually across various malignancies, including esophageal cancer (14–18). However, to our knowledge, no prior study has combined these two indices to assess their synergistic prognostic value in the neoadjuvant setting for ESCC. Because GRIm and CONUT capture different yet complementary aspects of host biology—systemic inflammation, immune competence, metabolic status, and nutritional reserve—their integration provides a more comprehensive reflection of the host–tumor interaction than either score alone (10–13, 15, 16, 19). These findings align with the concept that multi-parameter integration better reflects the complex interplay between tumor biology and host systemic status than single markers.

The biological rationale underlying the predictive value of the GRIm–CONUT score is also supported by mechanistic insights. Elevated LDH reflects tumor hypoxia and metabolic reprogramming, both of which promote tumor aggressiveness. Increased NLR suggests neutrophil-driven inflammation and compromised lymphocyte-mediated antitumor immunity (20–23). This condition results in depletion of key effector immune cells, particularly CD8^+^ cytotoxic T lymphocytes and natural killer (NK) cells, thereby compromising effective antitumor immune responses (24, 25). Accordingly, lymphopenia in cancer patients has been consistently linked to impaired tumor immune surveillance and diminished responsiveness to immunotherapy. Low albumin indicates a combination of malnutrition and systemic inflammation, while decreased cholesterol and lymphocyte counts further highlight impaired immune response and metabolic dysfunction (17, 26). these abnormalities may reduce the efficacy of neoadjuvant therapy, limit the host’s ability to mount an antitumor response, and contribute to worse survival outcomes. A favorable baseline GRIm–CONUT score is generally indicative of preserved lymphocyte reserves, reduced systemic inflammation, and relatively stable nutritional status. This intact immune–nutritional milieu may support more effective antitumor immune activity during treatment with immune checkpoint inhibitors (ICIs). In line with this concept, consistent prognostic trends were observed in the subgroup of patients treated with neoadjuvant chemoimmunotherapy. Clinically, the GRIm–CONUT score offers several advantages. It is simple, inexpensive, objective, and based entirely on routinely available laboratory parameters, making it easily applicable in diverse clinical settings. The strong association between higher GRIm–CONUT scores and reduced pCR rates suggests that this composite index may help identify patients less likely to benefit from standard neoadjuvant regimens. Our subgroup analysis further showed that, across all neoadjuvant treatment regimens, patients with a higher GRIm-CONUT score (2 vs. 0) exhibited a significantly higher risk of poor survival compared with those with a lower score. Moreover, the GRIm–CONUT–based nomogram demonstrated superior predictive performance compared with ypTNM staging alone, supporting its potential value in postoperative prognostic evaluation, risk stratification, treatment decision-making, and individualized surveillance planning.

Despite its strengths, this study has limitations. First, the retrospective, single-center design may introduce selection bias, and external validation is needed to confirm the generalizability of our findings. Second, laboratory indicators were collected at a single time point before treatment, preventing assessment of dynamic immune–nutritional changes during or after neoadjuvant therapy, which may also have prognostic implications (8, 27). Third, differences in neoadjuvant regimens and patient selection criteria across institutions may influence the cutoff values and prognostic performance of the GRIm–CONUT score. Notably, the prognostic performance of the GRIm–CONUT score may vary across different therapeutic strategies, particularly in the setting of neoadjuvant chemoimmunotherapy, and therefore warrants further investigation. In addition, because the number of patients achieving pathologic complete response (pCR) in our cohort was limited, analyses regarding the predictive value of GRIm–CONUT for pCR should be interpreted with caution. Larger, well-designed studies are required to validate these findings and to clarify the role of GRIm–CONUT across distinct treatment modalities.

Future research should include prospective, multicenter validation and standardized thresholds for GRIm–CONUT classification. Studies examining whether interventions targeting immune–nutritional status—such as nutritional optimization, anti-inflammatory strategies, or immunometabolic modulation—can improve treatment response and survival are warranted. Additionally, exploring dynamic GRIm–CONUT changes during treatment may further refine prognostic accuracy and guide personalized therapy.

## 5 Conclusion

The pretreatment GRIm–CONUT composite score, integrating systemic immune–inflammatory and nutritional status, is an independent predictor of pathologic response, overall survival, and recurrence-free survival in patients with ESCC undergoing neoadjuvant therapy followed by surgery. The GRIm–CONUT–based nomogram demonstrated superior prognostic accuracy and clinical utility compared with conventional ypTNM staging alone, highlighting its potential value in individualized risk stratification and postoperative management. As a simple, inexpensive, and readily accessible biomarker, the GRIm–CONUT score may aid in optimizing treatment strategies and identifying patients at elevated risk of poor outcomes. Prospective, multicenter studies are warranted to validate these findings and explore whether improving immune–nutritional status can enhance therapeutic efficacy in ESCC.

## Data Availability

The original contributions presented in the study are included in the article/supplementary material. Further inquiries can be directed to the corresponding authors.
